# Coronary microcirculation dysfunction causing ischemia with non-obstructive coronary arteries: a case report

**DOI:** 10.3389/fcvm.2025.1556064

**Published:** 2025-04-15

**Authors:** Wei Qi, Yazheng Zhang, Le Wang, Kai Hou, Ting Li, Jiachun Lang, Hongliang Cong

**Affiliations:** ^1^ Department of Cardiology, Tianjin Chest Hospital, Tianjin, China; ^2^ Department of Cardiology, Chest Hospital, Tianjin University, Tianjin, China; ^3^ Research Center, Pu’er People’s Hospital, Pu'er City, Yunnan, China

**Keywords:** coronary arteries, microcirculation dysfunction, myocardial ischemia, angina, variation

## Abstract

The study presents a case of INOCA attributed to CMVD in a 53-year-old male patient experiencing exertional angina, despite the absence of significant coronary artery stenosis on angiography. The patient presented with reversible myocardial ischemia detected by myocardial perfusion imaging, with an ischemic area accounting for 12% of the left ventricular wall. Diagnostic tests revealed an elevated index of microcirculatory resistance (IMR = 46.3) and a quantitative flow ratio (QFR = 0.94), confirming CMVD. Genetic testing identified a NOTCH1 c.3862G>A variant in the proband and some family members, suggesting a potential contribution to CMVD pathogenesis through impaired vascular remodeling and microcirculatory regulation. After six months of targeted treatment with nicorandil, coenzyme Q10, trimetazidine, and rosuvastatin, the patient's symptoms resolved, and myocardial ischemia reversed. While an MYH7 variant was also detected, its clinical relevance was ruled out due to the family's absence of associated cardiomyopathy phenotypes. The NOTCH1 gene may play a potential role in INOCA caused by CMVD, however, further research is needed to elucidate its underlying regulatory mechanisms. The findings provide a foundation for precise diagnosis and personalized management of INOCA.

## 1 Introduction

INOCA is defined as all coronary stenoses <50%, with the 2019 European Society of Cardiology guidelines noting that ∼45% of patients presenting with angina or ischemia showed no obstructive stable coronary artery disease based on angiography or stress testing (1, 2). CMVD may be a key cause of ischemia with non-obstructive coronary arteries (INOCA) (3). Coronary microvascular dysfunction (CMVD) refers to ischemic heart disease caused by abnormalities in the structure and function of the coronary microcirculation. Among the 112 million angina patients worldwide, approximately 70% of them show no abnormalities upon coronary angiography but experience symptoms of coronary artery disease (4). The typical symptoms of CMVD include effort-induced angina, and in resting conditions, patients may also experience atypical symptoms such as pressure-like discomfort or intermittent chest pain (5).

Although experimental studies show that CMVD can induce myocardial ischemia, its role in ischemic syndromes remains unclear in clinical practice. This is due to the inability of angiography to visualize small coronary artery abnormalities, the need for complex and time-consuming methods to assess microcirculation function invasively, and the lack of detectable signs of ischemia, such as stress-induced left ventricular contractile changes and ischemic metabolites, in patients suspected of having microvascular angina (6). The primary structural changes in coronary microvascular dysfunction include luminal narrowing of arterioles and capillaries, perivascular fibrosis, and capillary rarefaction, often associated with left ventricular hypertrophy, hypertrophic cardiomyopathy, and hypertensive heart disease, leading to impaired coronary blood flow (7). Over 50% of ischemic heart disease susceptibility is linked to genetic variants, primarily single-nucleotide polymorphisms identified through genome-wide association studies, which can predispose individuals to CMVD, leading to ischemia and myocardial infarction in non-obstructive coronary arteries by affecting proteins involved in coronary blood flow regulation (8).

This paper reported a case of INOCA caused by microcirculation disturbance, diagnosis, treatment and follow-up, and genetic study of the proband and her family members.

## 2 Case report

A 53-year-old male patient presented to our hospital in January 2024 with intermittent chest pain in the anterior chest region following exertion, radiating to the left shoulder, left arm, and back. The pain was accompanied by sensations of choking, chest tightness, and a feeling of obstruction. Upon inquiry, the symptoms were found to be activity-related, occurring after walking approximately 100 m, with episodes lasting 3–5 min and occurring frequently. The patient was admitted for further diagnosis and treatment.

**Figure 1:**
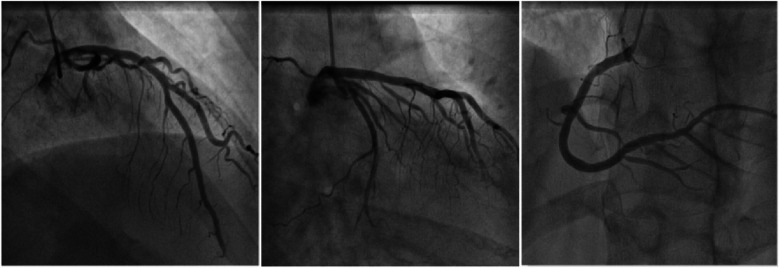
Schematic diagram of coronary angiography. From left to right are the left anterior descending artery, circumflex artery, and right coronary artery.

**Figure 2:**
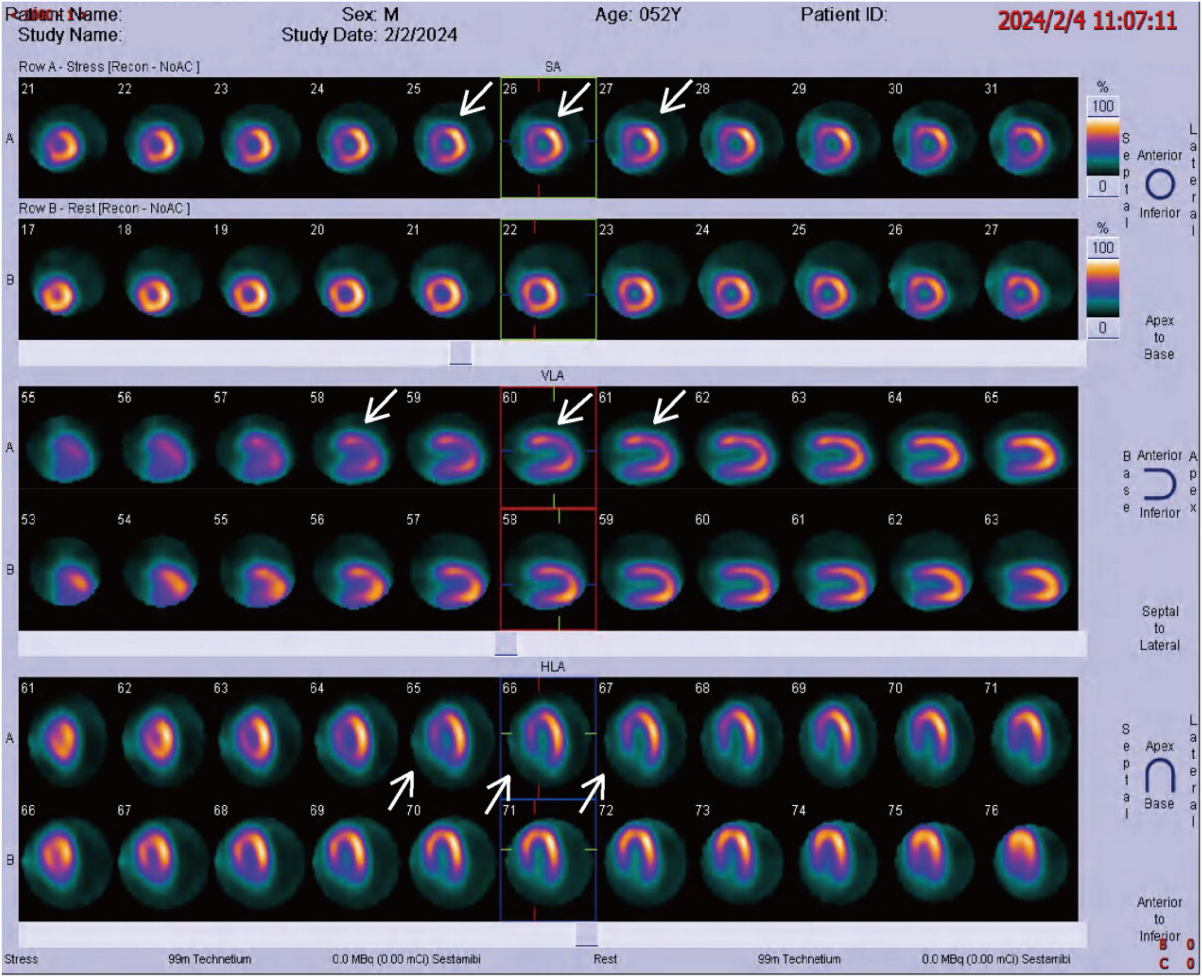
Radionuclide myocardial perfusion imaging, with white arrows indicating myocardial ischemia.

Physical examination on admission revealed a heart rate of 78 bpm and blood pressure of 127/71 mmHg. An electrocardiogram showed sinus rhythm, ST-T wave changes, and left bundle branch block, indicating myocardial ischemia. Echocardiography revealed aortic sclerosis, mild regurgitation of the mitral and tricuspid valves, normal cardiac structure, and a normal left ventricular ejection fraction. The patient had significant angina symptoms classified as Grade III according to the Canadian Cardiovascular Society Angina Grading Scale (CCS). Coronary angiography indicated mild coronary stenosis <50% (Figure 1). Myocardial perfusion imaging showed mild to moderate reversible myocardial ischemia in a portion of the left ventricular apex and part of the interventricular septum, involving approximately 12% of the left ventricular wall. Based on coronary angiography and imaging results, the patient exhibited slight enlargement of the left ventricular cavity during stress imaging compared to rest imaging, with a decrease in the left ventricular ejection fraction, suggesting a possible association with microcirculatory dysfunction (Figure 2).

**Figure 3:**
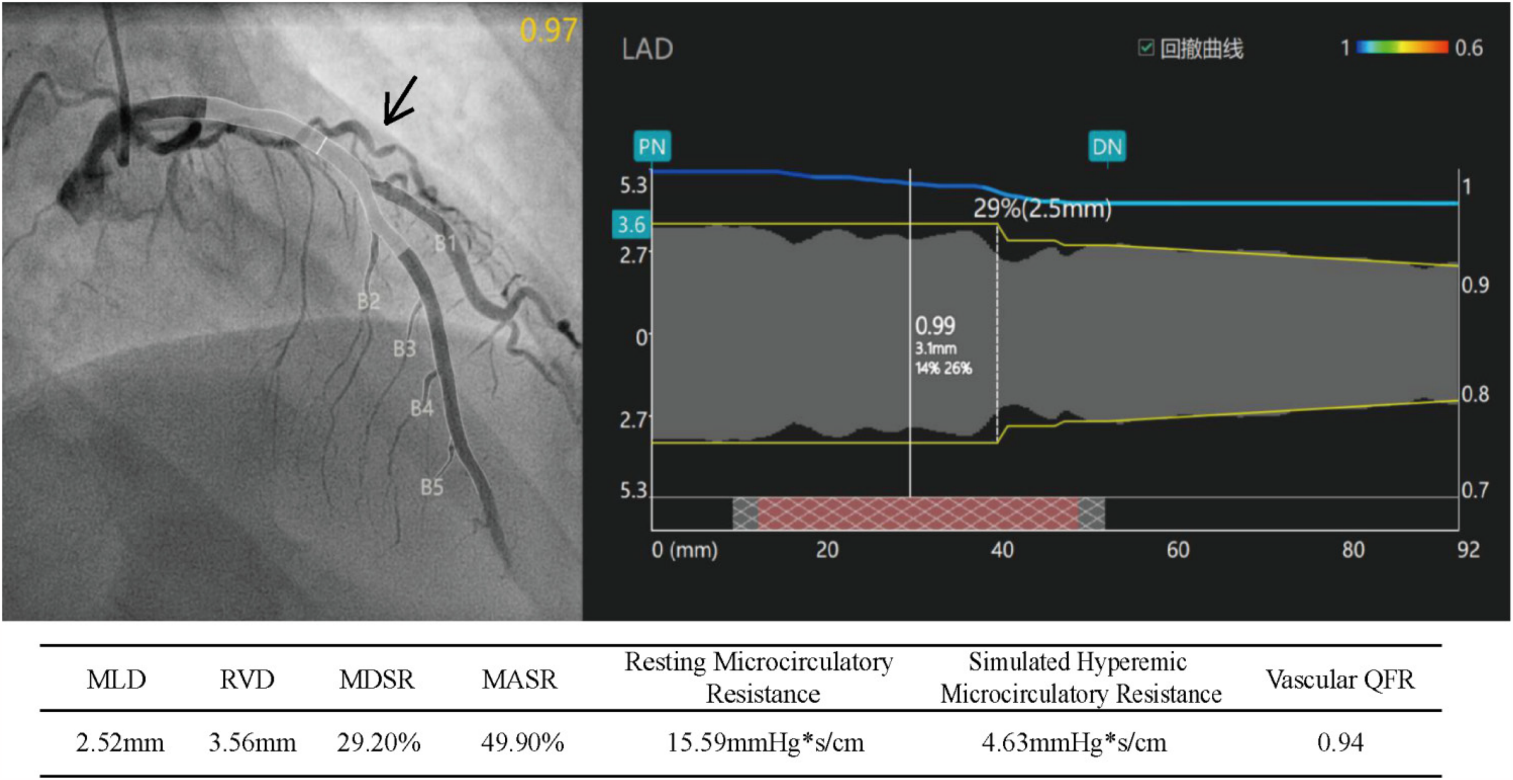
Index of microcirculatory resistance (IMR) and quantitative flow reserve (QFR) of the left anterior descending artery (IMR = simulated hyperemic microcirculatory resistance × 10). MLD, minimum lumen diameter; RVD, reference vessel diameter; MDSR, maximum diameter stenosis rate; MASR, maximum area stenosis rate.

Subsequently, coronary microcirculatory resistance index (IMR) and quantitative flow ratio (QFR) testing were performed. The stenosis rate of the left anterior descending (LAD) artery ranged from 22.5% to 29.2%, all <50%. The QFR of the LAD was 0.94, and the IMR was 46.3, well above the threshold of 25, indicating coronary microvascular dysfunction (CMVD) (Figure 3).

Additionally, to rule out myocardial disease as the cause of the patient's chest tightness and other discomforts, cardiac MRI with contrast enhancement was performed. The left heart function and structure showed no significant abnormalities, no obvious myocardial fibrosis, and a low left ventricular T1 mapping value (1,172 ± 80 ms). The patient was treated with oral nicorandil (5 mg, three times daily), coenzyme Q10 (10 mg, three times daily), trimetazidine (20 mg, three times daily), and rosuvastatin (5 mg, once in the evening). The patient was prescribed 5 mg of rosuvastatin to provide minimal vascular protection while avoiding unnecessary risks, given their normal lipid levels, lack of significant coronary stenosis, absence of inflammatory or oxidative stress markers, and the need to minimize potential side effects. Additionally, aspirin was not administered to the patient due to the lack of a coronary artery disease diagnosis, the suspected presence of microvascular dysfunction based on the IMR index, and concerns regarding its potential side effects, such as platelet inhibition and an increased risk of bleeding. Six months later, the patient had no significant angina symptoms, and a follow-up myocardial perfusion scan showed no significant reversible ischemia (Supplementary Figure S1).

The proband's father (II-2) has a history of coronary artery disease and heart failure and was treated at our hospital's outpatient clinic. His electrocardiogram showed atrial arrhythmia, poor R-wave progression in the precordial leads, and ST-T changes. The patient has heart failure with New York Heart Association (NYHA) Functional Class IV, with suspected ischemic cardiomyopathy. Contrast-enhanced CTA revealed left main and three-vessel coronary artery disease, and ultrasound indicated aortic valve calcification with reduced ejection fraction.

I-1 (the proband's grandmother) died at age 60 from lung cancer, and I-2 (the proband's grandfather) died suddenly at age 62, possibly from a myocardial infarction. III-3 (the proband's brother) is asymptomatic, and his electrocardiogram showed no abnormalities. IV-1 (the proband's son) had a normal electrocardiogram (Supplementary Figure S2).

**Figure 4:**
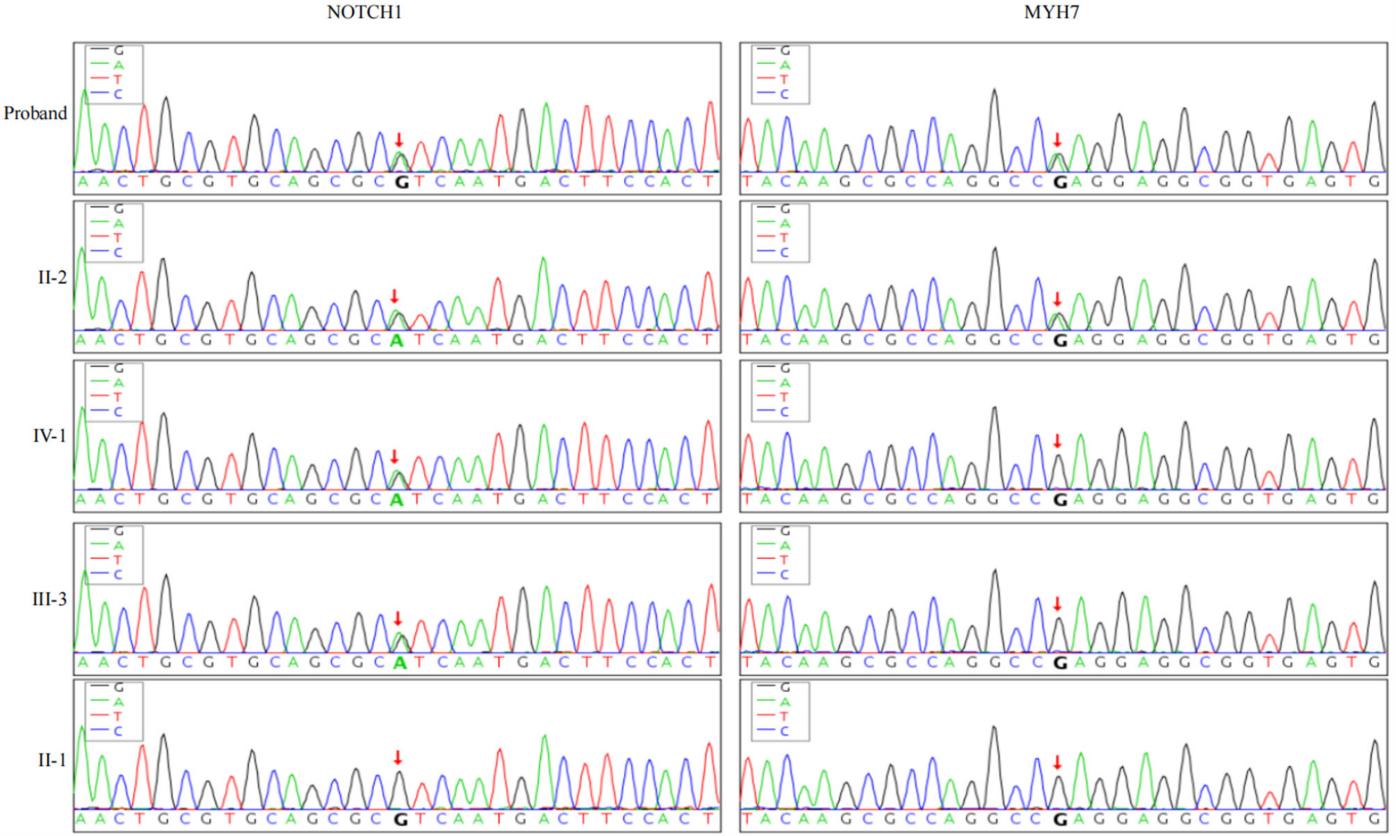
Genetic sequencing results of NOTCH1 and MYH7 genes in family members: the proband and his father carry heterozygous variants NOTCH1 c.3862G>A and MYH7 c.5647G>A. The proband's son and brother carry only the heterozygous NOTCH1 c.3862G>A variant, while the proband's mother has wild-type alleles for both genes.

The proband's grandparents have already passed away, so blood samples could not be obtained and were therefore excluded from the analysis. Exome sequencing of family members revealed a novel heterozygous mutation (c.5647G>A p.Glu1883Lys) in exon 38 of the MYH7 gene on chromosome 14, which is a missense mutation, present in both II-2 and the proband. Additionally, a novel heterozygous mutation (c.3862G>A p.Val1288Ile) was found in exon 23 of the NOTCH1 gene, present in II-2, III-2, III-3, and IV-1, which is also a missense mutation (Figure 4). According to the ACMG guidelines, the MYH7 gene mutation was classified as PM2_Supporting + PP3, and the NOTCH1 mutation was classified as PM2_Supporting.

## 3 Discussion

Ischemic heart disease is a leading cause of mortality and morbidity worldwide. In clinical practice, patients with coronary artery disease (CAD) may show normal or non-obstructive coronary arteries upon coronary angiography, which could be attributed to coronary microvascular dysfunction (CMVD) (9). According to the guidelines of the European Society of Cardiology (ESC), invasive methods such as Coronary Flow Reserve (CFR) and IMR play a crucial role in assessing coronary microvascular function, especially in patients with INOCA (10). Canu M. et al. highlighted that IMR and HMR are essential for the early detection of severe CMVD in STEMI patients, serving as important prognostic markers and potential therapeutic targets for clinical intervention (11). It is currently believed that patients with coronary microvascular dysfunction are at higher risk of developing hypertrophic cardiomyopathy, dilated cardiomyopathy, Fabry disease, aortic valve stenosis, and stable angina (12). Exogenous microvascular dysfunction caused by myocardial cell hypertrophy, cardiomyopathy, or left ventricular diastolic dysfunction may play a crucial role in the initiation of myocardial ischemia or necrosis (13). In our case, despite the presence of coronary artery stenosis, the stenosis rate was less than 50%, which could not explain the coronary ischemia and angina symptoms. However, based on myocardial scintigraphy, cardiac MRI, and stress imaging, we speculate that the symptoms may be due to a combination of microvascular dysfunction and coronary artery stenosis. The patient's left anterior descending (LAD) coronary artery QFR was 0.94, and the microvascular resistance index (IMR) was 46.3, much higher than the diagnostic threshold of IMR > 25 (14, 15), with increased microvascular resistance in the LAD territory corresponding to the myocardial ischemia seen on the scintigraphy. This suggests that the myocardial ischemia in this patient was caused by coronary microvascular dysfunction, which was significantly reversed after six months of treatment aimed at improving coronary microcirculation.

Although genetic testing identified MYH7 gene mutations in both the proband and his father, these mutations are primarily associated with hypertrophic cardiomyopathy and dilated cardiomyopathy (16, 17), and neither the proband nor his family members exhibited related clinical symptoms. Thus, we exclude the pathogenic effect of this mutation. Mutations in the NOTCH1 gene are known to cause aortic valve stenosis and calcific atrioventricular disease, which are important factors in the development of heart disease (18). Activation of NOTCH1 in myocardial cells is closely related to myocardial injury repair (19), and binding of NOTCH1 signaling proteins to Jagged1 reduces fibrotic tissue proliferation, increases microvascular density, and inhibits cardiac endothelial-mesenchymal transition (EMT), thereby preventing myocardial fibrosis (20). While activation of the Notch signaling pathway promotes Nrg-1 transcription and inhibits endothelial cell apoptosis by facilitating NICD binding to histone acetylase GCN5, forming a regulatory complex that enhances GCN5 recruitment to the Nrg-1 promoter, modulates histone acetylation via H3K9Ac, and regulates myocardial microvascular endothelial cell function to mitigate coronary microvascular dysfunction (21). The VEGFA-NOTCH1 signaling pathway can regulate trabecular blood vessel regeneration during EMT transformation, which helps prevent trabecular myocardial damage and promotes normal coronary microcirculation (22). Therefore, based on the phenotypes of the proband and his father, we infer that the heterozygous mutation of NOTCH1 c.3862G>A may be an important factor in the development of CMVD in the family. The differences in the copy number variation of the NOTCH1 gene and the resulting gene dosage effect (23, 24) may explain why the patient's brother and son have no symptoms yet. Although there are reports on the relationship between the NOTCH1 signaling pathway and aortic valve lesions, valve calcification, and atrioventricular fibrosis (25), as well as its close involvement in coronary artery development and migration (26), the relationship and regulatory mechanisms of the NOTCH1 signaling pathway in endothelial dysfunction are still unclear.

The findings of this study suggest that the NOTCH1 signaling pathway may play a potential role in regulating vascular network remodeling and warrants further basic research to clarify the exact regulatory mechanisms. Due to the limited number of family members, we need more cases and larger families to confirm the role of NOTCH1 gene variants in INOCA caused by CMVD.

## Data Availability

The datasets generated and/or analyzed during this study are available from the corresponding author upon reasonable request.
