# Supplementary material for: Coronary microcirculation dysfunction causing ischemia with non-obstructive coronary arteries: a case report
Source: Front Cardiovasc Med. 2025 Apr 15;12:1556064. doi: 10.3389/fcvm.2025.1556064 (PMC12037479; doi:10.3389/fcvm.2025.1556064)
Supplement: Supplementary file 1 [file Datasheet1.pdf]

Supplementary Material

1 Supplementary Figures

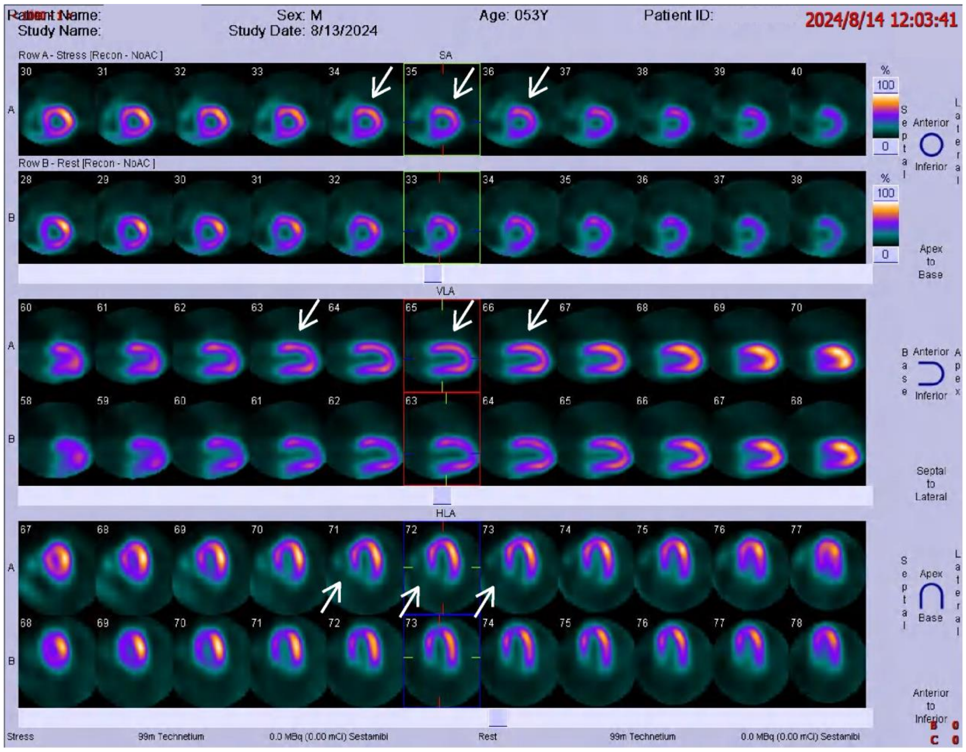

Supplementary Figure 1. Radionuclide myocardial perfusion imaging after six months of treatment.

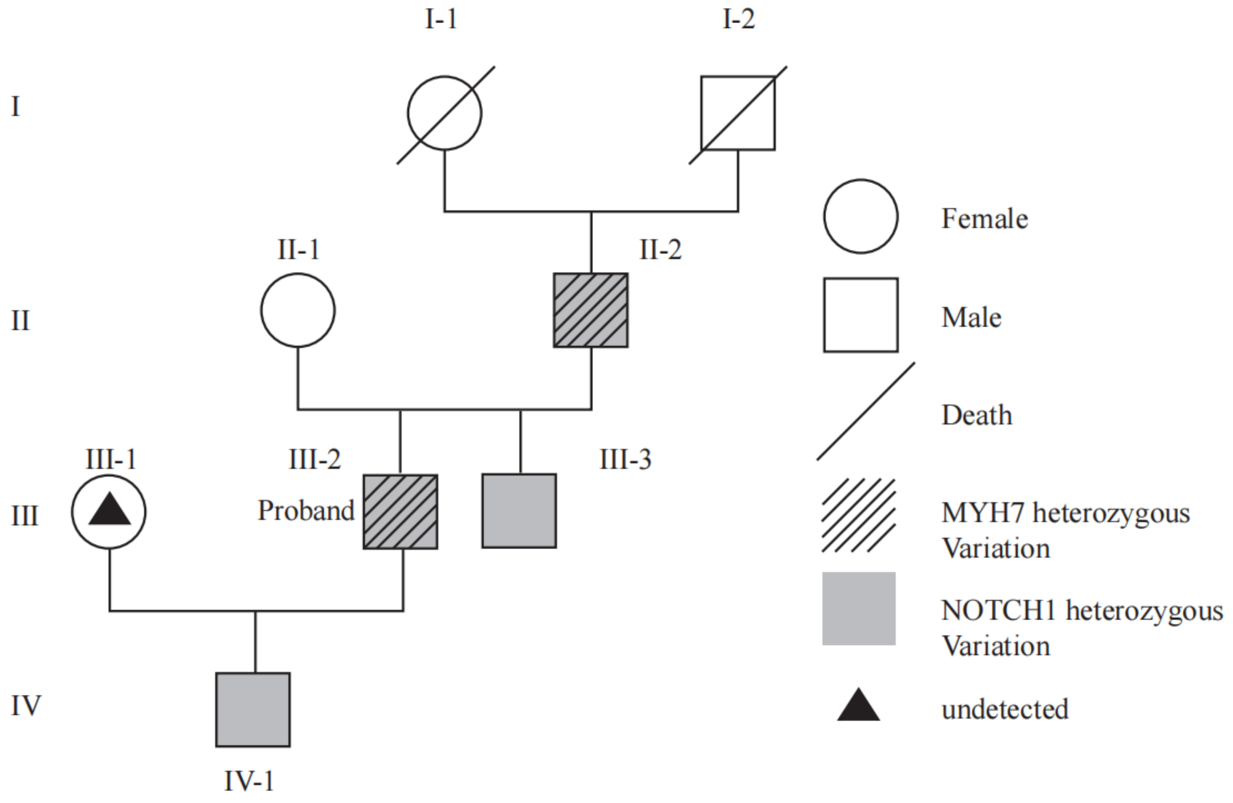

Supplementary Figure 2. Investigation of the proband and family members. The proband (III-2) and his father (II-2) carry heterozygous variants in the NOTCH1 and MYH7 genes, while III-3 and IV-1 only carry a heterozygous variant in the NOTCH1 gene.
